# Effects of water stress on nutrients and enzyme activity in rhizosphere soils of greenhouse grape

**DOI:** 10.3389/fmicb.2024.1376849

**Published:** 2024-03-18

**Authors:** Rui Zhang, Hongjuan Zhang, Changyu Yang, Hongxia Li, Jiangqi Wu

**Affiliations:** ^1^College of Water Conservancy and Hydropower Engineering, Gansu Agricultural University, Lanzhou, China; ^2^College of Forestry, Gansu Agricultural University, Lanzhou, China

**Keywords:** water stress, physical–chemical properties, microbial biomass, enzyme activity, greenhouse grape

## Abstract

In grape cultivation, incorrect water regulation will lead to significant water wastage, which in turn will change soil structure and disrupt soil nutrient cycling processes. This study aimed to investigate the effects of different water regulation treatments [by setting moderate water stress (W1), mild water stress (W2), and adequate water availability (CK)] on soil physical–chemical properties and enzyme activity in greenhouse grape during the growing season. The result showed that the W2 treatment had a negative impact on the build-up of dissolved organic carbon (DOC), nitrate nitrogen (NO_3_-N), and available phosphorus (AP). Throughout the reproductive period, the W1 and W2 treatments decreased the soil’s microbial biomass carbon (MBC) and microbial biomass nitrogen (MBN) contents, and MBC was more vulnerable to water stress. During the growth period, the trends of urease, catalase, and sucrase activities in different soil depth were ranked as 10–20 cm > 0–10 cm > 20–40 cm. The urease activity in 0–10 cm soil was suppressed by both W1 and W2 treatments, while the invertase activity in various soil layers under W1 treatment differed substantially. The W1 treatment also reduced the catalase activity in the 20–40 cm soil layer in the grape growth season. These findings suggested that W2 treatment can conserve water and enhance microbial ecology of greenhouse grape soils. Therefore, W2 treatment was the most effective water regulation measure for local greenhouse grape cultivation.

## 1 Introduction

Water scarcity is the most main environmental stress for crop growth, and water stress-induced agriculture failure will cause serious ecological and food security issues (Yang et al., 2021; Zhang C. et al., 2023). Water stress not only affected crop growth and microbial structure, limits soil nutrient transport but also leaded to a significant decrease in soil microbial biomass (Manzoni et al., 2012). The rhizosphere is the site of plant–soil-microorganism interaction, and the soil area where plants are highly sensitive to external environmental stress (Zhang et al., 2019). Studies have shown that water stress can impact crop chlorophyll levels (Ru et al., 2020), metabolic processes (Kapoor et al., 2020), root growth (Wang et al., 2019), and soil microbial communities (Bogati and Walczak, 2022). Secretions released during crop growth period also changed soil enzyme activity (Xiao et al., 2023). Therefore, complex interactions between soil water and crops combine to influence changes of rhizosphere soil microbials.

Physical–chemical property and microbial biomass are important regulators of soil element cycling and crop nutrient supply, and are the most sensitive and potential soil biological indicators (Marschner et al., 2005; Zhang et al., 2016; Yang et al., 2019). Soil organic carbon (SOC), total nitrogen (TN), total phosphorus (TP), nitrate nitrogen (NO_3_-N), ammonia nitrogen (NH_4_-N), and availability phosphorus (AP) are the main nutrients in soil and are crucial to all biological processes (Wu et al., 2020). Water stress severely disrupted the structure and function of soil, soil microbial biomass reaches 14.3%, and soil moisture is maintained above 10%, which in turn avoided damaging the soil system (Geng et al., 2015). Water stress not only decreases soil chemical fertility but also reduces microbial activity, including the activities of invertase and urease (Wu et al., 2012). The plant root system will firstly experience water stress, mainly as a result of inadequate or excessive water in the soil (Kim et al., 2020). Soil moisture affects the transfer and transportation of soil nutrients, which in turn affects the growth and reproduction of microorganisms (Xue et al., 2017). Therefore, the investigation of the impact of water stress on rhizosphere soil moisture, nutrients, and microorganisms in plants were critical.

Soil enzymes are specialized proteins with biocatalytic activity, known as “active reservoirs of plant nutrients.” Enzyme activity is higher in rhizosphere soil than in bulk soil (Marschner et al., 2005). In addition, catalase, urease, and sucrose were the main environmental variables affecting the composition of the soil microbial community in the grape rhizosphere (Song et al., 2024). Soil enzyme activity increased with increasing soil moisture, but decreased with excess soil water, as shown by Chrost (2014). Gramss et al. (1999) also found that adequate water stress (80% field water holding capacity) could stimulate plant roots to produce more enzymes, and this was due to oxygen limiting microbial activity. Soil enzyme activities were related to soil temperature, physical–chemical properties, and pH, respectively, and varied in different planting types and growth stages (Barta et al., 2014; Menichetti et al., 2015; Liu and Zhang, 2019; Jing et al., 2020; Zhao et al., 2020). In addition, some studies have shown that water stress can increase soil peroxidase, phosphatase, dehydrogenase, saccharase, and phosphatase activities (Song et al., 2012; Padhy et al., 2018; Kátai et al., 2020). However, the response of soil enzyme activities to water stress remains highly uncertain. Therefore, the aim of this study was to analyze the changes in rhizosphere soil enzyme activities of greenhouse grapes by different water stresses, which could further characterize the changes in enzyme activities.

In this study, the rhizosphere soil of greenhouse grapes in arid area was the object of study, and different water stress treatments were established. We analyzed the physical–chemical properties, microbial biomass, and enzyme activities of the soil to provide a theoretical basis for the scientific cultivation of greenhouse grapes in the arid region. The objectives of the study were: (1) to analyze the effects of different water stress conditions on physical–chemical properties, microbial biomass, and enzyme activities in rhizosphere soil at different growth stages; and (2) to explore the correlation between soil physical–chemical properties, microbial biomass and enzyme activity.

## 2 Materials and methods

### 2.1 Study site

The field experiment was conducted in 2019 at the Yongdeng Irrigation Experiment Station (36°43′34″N; 103°16′24″E; altitude: 2100 m) in Gansu Province, China. The study area is located in a semi-arid region, with a typical continental monsoon climate, and the average annual rainfall, evaporation, and temperature were 290 mm, 1,000 mm, and 5.9°C, respectively. The soil type of the experimental field was mainly loam, the water capacity was 29.2%, the density was 1.42 g cm^−3^, and the soil pH was 8.15.

### 2.2 Experimental design and management

*Red globe*, a 5-year-old Eurasian grape variety, was used as the test material. Grapes were planted in a plastic greenhouse of 8 m × 80 m with an earth wall straw curtain and the cultivation method of a single-arm Y-shaped low single-hedge frame was adopted. The row spacing was 2.0 m, and the plant spacing was 0.8 m. Each row (each treatment) comprised eight grapes, and the row direction was perpendicular to the greenhouse direction (Zhang et al., 2019). The experimental plots were designed in a randomized block group design and there were three replications for each treatment, containing a total of nine plots with a plot size of 8 m × 2 m.

The growth period of grapes was divided into five stages based on local protected-cultivation grape water consumption and irrigation experience (Table 1). The experiment included three treatments: (1) W1 treatment was moderate water stress (lower limit of soil moisture content was 55%); (2) W2 treatment was mild water stress (lower limit of soil moisture content was 65%); (3) CK treatment was the control treatment (sufficient water supply). The field trial of greenhouse grapes was irrigated by drip irrigation with the control mode of “one pipe and one row,” and the flow rate of sprinkler head was 3 L H^−1^. The irrigation was performed when the soil moisture in the field trial reached the lower limit of the experimental design, and the irrigation rate was 270 m^3^ hm^−2^. The valves and the water meters installed in each plot were used to control the amount of irrigation. The irrigation amount and irrigation time were determined by the soil moisture content and measured using a water meter. The soil moisture ratio was 0.5, and the planned depth of the wetting layer was 80 cm.

**Table 1:** Field experiment design for greenhouse grapes.

Growth stage	Treatment		
	W1	W2	CK
	Lower limit of water content/%	Lower limit of water content/%	Lower limit of water content/%
Budburst stage (May 15–May 24)	55	65	75
New shoot elongation stage (May 25–June 22)	55	65	75
Flowering stage (June 23–July 15)	55	65	75
Fruit enlargement stage (July 16–September 22)	55	65	75
Coloring maturity stage (September 25–October 22)	55	65	75

Local farming management practices were referred to for the use of fertilization, and insecticide and herbicide management. On 24th February, basal fertilizer (chicken manure; 5,000 kg hm^−2^) was applied along with 2 kg diammonium phosphate and 4 kg ammonium bicarbonate. Each treatment received 1 kg diammonium phosphate, 0.8 kg calcium ammonium nitrate for agriculture, 0.8 kg of organic fertilizer, and 0.5 kg of potassium magnesium sulfate at the 16th June, respectively. On 16th August, 0.8 kg diammonium phosphate, 0.8 kg calcium ammonium nitrate for agriculture, 0.8 kg organic fertilizer, and 0.6 kg potassium and magnesium sulfate were applied.

### 2.3 Collection of rhizosphere soil sample

Soil samples (0–40 cm) were collected on May 15, June 15, July 20, August 15, and October 15, 2019 at different growth periods. Three soil cores (3 cm diameter) of grape rhizosphere soil in each plot were taken at a depth of 0–10, 10–20, and 20–40 cm, respectively. Then, the samples in each plot were pooled by depth and transported to the laboratory. In the lab, the samples were sieved through a 2 mm mesh. The soil layer of 10–20 cm was divided into three parts; one part was air-dried at room temperature for determining some basic physical–chemical indices of the soil, and the other part was stored in a refrigerator at 4°C for fresh sample analysis (partial basic physical–chemical indices of soil and enzyme activity). Sterile gloves were worn during the sampling process. The ziplock bag, spatula, and other tools used for sampling were sterilized at high temperature to eliminate test errors. The measurement of each index was completed within 1 month after the completion of the sampling.

### 2.4 Soil physical–chemical properties

Total nitrogen (TN), nitrate nitrogen (NO_3_-N), and ammonia nitrogen (NH_4_-N) contents were determined by the Kjeldahl method (Boell and Shen, 1954) (Table 2). The soil total organic carbon (TOC) content was measured using a carbon and nitrogen combined analyzer (Multi N/C 2100 s, Jena, Germany) after removing inorganic carbon with 0.5 mol/L dilute hydrochloric acid. The dissolved organic carbon (DOC) content was determined with a carbon and nitrogen combined analyzer (Multi C/N 2100 s) after leaching with ultrapure water (water: soil = 5: 1). The microbial biomass carbon (MBC) and microbial biomass nitrogen (MBN) content were determined using a carbon and nitrogen combined analyzer after 0.5 mol·L^−1^ K_2_SO_4_ extraction (Multi C/N 2100 s) (Xu et al., 2016).

**Table 2:** Parametric determination of physical–chemical properties of greenhouse grape rhizosphere soils.

Index	Determination method
TN (mg kg^−1^), NO_3_-N (mg kg^−1^), and NH_4_-N (mg kg^−1^)	The Kjeldahl
TOC (g kg^−1^), DOC (mg kg^−1^), MBC (mg kg^−1^), and MBN (mg kg^−1^)	The carbon and nitrogen combined analyzer
Soil sucrase activity (mg g^−1^ 24 h^−1^)	The 3–5 dinitrosalicylic acid method
Soil catalase activity (mg g^−1^ 24 h^−1^)	The KMnO_4_ liquid titration method
Soil urease activity (mg g^−1^ 24 h^−1^)	The indophenol blue colorimetry method

TN, Total nitrogen; NO_3_-N, Nitrate nitrogen; NH_4_-N, Ammonia nitrogen; TOC, Total organic carbon; DOC, Dissolved organic carbon; MBC, Microbial biomass carbon; and MBN, Microbial biomass nitrogen.

To determine the sucrase activity, 5 g of air-dried soils were incubated for 24 h at 37°C with 15 mL of 8% sucrose, 5 mL of phosphate buffer at pH 5.5, and 0.1 mL of toluene. The glucose released by sucrase reacted with 3-5-dinitrosalicylic acid and then was measured based on the absorbance at 508 nm (UV-2450, Shimadzu Corporation, Kyoto, Japan) (Wu et al., 2020). The results were expressed as mg glucose g^−1^⋅h^−1^. Soil urease activity was determined by indophenol blue colorimetry and expressed as mg of NH_3_-N in 5 g air-dried soil after incubating for 24 h at 37°C with 20 mL of citrate buffer at pH 6.7, 10 mL of 10% urea, and 0.1 mL of toluene (Xie et al., 2017). Soil catalase activity was determined by the KMnO_4_ liquid titration method and expressed as the volume of 0.02 mol·L^−1^ KMnO_4_ consumed of 2 g air-dried soil within 20 min (Li et al., 2014).

### 2.5 Statistical analysis

The relationships between soil microbial biomass, enzyme activities and soil physical–chemical properties were analyzed using Spearman’s correlation method (SPSS 27.0). Correlations among soil physical–chemical properties, enzyme activities, and microbial biomass were assessed using Person correlation analysis. One-way ANOVA was used to investigate the data for the different water stress treatments (*p* < 0.05).

## 3 Results

### 3.1 Effect of water stress on basic physical–chemical properties of greenhouse grape rhizosphere soil

**Figure 1:**
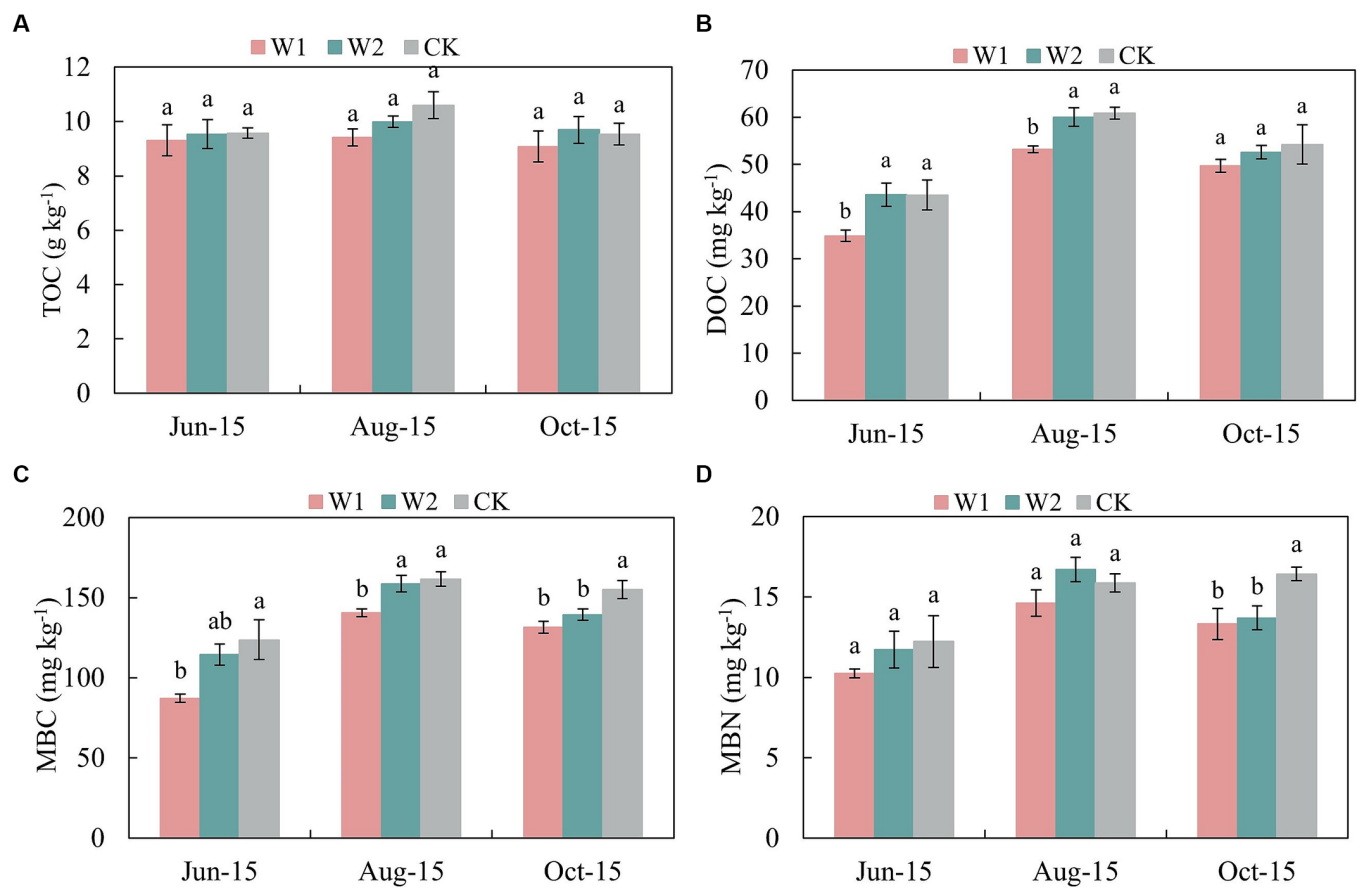
Effects of water stress on the TOC, DOC, MBC, and MBN content of greenhouse grape rhizosphere soil. Error bars indicate standard errors of the mean (*n* = 3). Different lowercase letters indicate significant differences among treatments (*p* < 0.05). W1, Moderate water stress (55% of field capacity); W2, Mild water stress (65% of field capacity); CK, Sufficient water supply (75% of field capacity). TOC, Total organic carbon; DOC, Dissolved organic carbon; MBC, Microbial biomass carbon; and MBN, Microbial biomass nitrogen. **(A)** is TOC content; **(B)** is DOC content; **(C)** is MBC content; **(D)** is MBN content.

Compared with CK treatment, the NO_3_-N content of W1 and W2 treatments decreased by 17.99, 16.07, 16.85, and 8.94% at the new shoot elongation and fruit enlargement stage, respectively (Table 3). The soil AP content showed an increasing and then stabilizing trend. In addition, the soil AP content was significantly (*p* < 0.05) higher in the CK treatment than in the W1 treatment during both shoot elongation and fruit enlargement stage. Thus, the AP content in the soil had specific adaptability to water stress. Moreover, there was no significant difference of TOC content between W1 and W2 treatment (Figure 1A). The soil DOC content showed an increasing trend and then decreasing during the growth period under different treatments, and peaked at the fruit enlargement stage (Aug-15) (Figure 1B). At the same time, compared with CK treatment, the DOC content of W1 treatment in maize growth stage was 19.84% (*p* < 0.05), 12.60% (*p* < 0.05) and 8.34% (*p* < 0.05) lower than that of CK treatment, respectively. Compared with CK treatment, the soil MBC content of W1 and W2 treatments was significantly lower by 5.61% (*p* < 0.05) and 15.63% (*p* < 0.05) at the coloring maturity stage (Oct-15), respectively (Figure 1C). With the increase duration of water stress, MBN was significantly lower in the W1 and W2 treatments than in the CK treatment (*p* < 0.05) when the coloring maturity stage was reached (Figure 1D).

**Table 3:** Changes in basic physical–chemical properties (mean ± standard deviation, *n* = 3) of soil under different water stresses.

Stage	Treatment	TN (mg kg^−1^)	NO_3_-N (mg kg^−1^)	NH_4_-N (mg kg^−1^)	TP (mg kg^−1^)	AP (mg kg^−1^)	SOM (mg kg^−1^)
Jun-15	W1	0.83 ± 0.03a	12.06 ± 0.97b	3.65 ± 0.76a	0.82 ± 0.09 a	28.88 ± 1.91b	16.03 ± 1.71a
	W2	0.82 ± 0.03a	12.26 ± 0.56b	3.42 ± 0.29a	0.79 ± 0.06 a	30.88 ± 1.88ab	16.44 ± 1.61a
	CK	0.83 ± 0.03a	14.23 ± 0.74a	3.88 ± 0.34a	0.77 ± 0.21 a	34.68 ± 2.50a	16.49 ± 0.57a
Aug-15	W1	0.83 ± 0.03a	15.85 ± 0.61b	4.50 ± 0.63a	0.83 ± 0.01 a	39.24 ± 1.08b	16.22 ± 0.95a
	W2	0.82 ± 0.03a	17.00 ± 1.18ab	4.47 ± 0.32a	0.84 ± 0.06 a	40.28 ± 1.63ab	17.21 ± 0.62a
	CK	0.81 ± 0.03a	18.52 ± 0.67a	4.88 ± 0.84a	0.79 ± 0.01 a	44.66 ± 3.45a	18.27 ± 1.47a
Oct-15	W1	0.84 ± 0.01a	12.49 ± 1.24a	3.53 ± 0.41a	0.82 ± 0.04 a	41.30 ± 1.63a	15.64 ± 1.68a
	W2	0.83 ± 0.01a	12.29 ± 0.76a	3.83 ± 0.38a	0.85 ± 0.09 a	37.66 ± 8.19a	16.71 ± 1.48a
	CK	0.82 ± 0.01a	13.80 ± 1.21a	3.57 ± 0.28a	0.84 ± 0.06 a	46.81 ± 2.15a	16.42 ± 1.19a

Different lowercase letters indicate significant differences among treatments (*p* < 0.05). W1, Moderate water stress (55% of field capacity); W2, Mild water stress (65% of field capacity); CK, Sufficient water supply (75% of field capacity). TN, Total nitrogen; NO_3_-N, Nitrate nitrogen; NH_4_-N, Ammonia nitrogen; TP, Total phosphorus; AP, Availability phosphorus; and SOM, Soil organic matter.

### 3.2 Effects of water stress on soil enzyme activities in greenhouse grape rhizosphere soil

Throughout the grape growing period, urease, catalase, and sucrase activities in different soil layers were ranked as: 10–20 cm > 0–10 cm > 20–40 cm (Table 4). Compared with the CK treatment, the urease activity in the 0–10 cm soil layer of W2 and W2 treatments was significantly lower by 27.40% (*p* < 0.05), 26.03% (*p* < 0.05), 20.69% (*p* < 0.05), and 44.87% (*p* < 0.05) at the new shoot elongation stage (Jun-15) and flowering stage (Jul-20), respectively. The urease activity in the 10–20 and 20–40 cm soil layers were significantly higher in the CK treatment than in the W1 treatment at the coloring maturity stage (Oct-15) (*p* < 0.05). The peroxidase activity of all soil layers of grapes decreased significantly during the period from budburst stage (May-15) to fruit enlargement (Aug-15). Compared with CK treatment, the peroxidase activity of W1 treatment in different soil layers (0–10, 10–20, and 20–40 cm) was significantly decreased by 3.65% (*p* < 0.05), 1.09% (*p* < 0.05), and 8.89% (*p* < 0.05) at budburst stage (May-15), respectively. With the increase of stress duration, the surface soil invertase activity of W1 treatment was significantly lower than that of CK treatment by 32.07% (*p* < 0.05) and 42.73% (*p* < 0.05) at the fruit enlargement stage (Aug-15) and coloring maturity stage (Oct-15), respectively. Furthermore, the conversion enzyme activity in the 20–40 cm soil layer under the W2 treatment was slightly higher than that in the W1 treatment during the period from the new shoot elongation stage (Jun-15) to coloring maturity stage (Oct-15). This result indicated that both W2 and W1 treatments inhibited soil urease, catalase, and sucrase activity convertase during the greenhouse grape growth.

**Table 4:** Effect of water stress on urease, catalase, and sucrase enzyme activity (mean ± standard deviation, *n* = 3) in the rhizosphere soil of delayed-cultivation greenhouse grapes (mg·g^−1^·day^−1^).

Enzyme	Soil depth/cm	Treatment	May-15	Jun-15	Jul-20	Aug-15	Oct-15
Urease	0–10	W1	0.64 ± 0.01b	0.53 ± 0.05b	0.46 ± 0.01b	0.52 ± 0.08b	0.39 ± 0.02b
		W2	0.74 ± 0.02a	0.54 ± 0.04b	0.42 ± 0.01b	0.83 ± 0.08a	0.59 ± 0.09a
		CK	0.54 ± 0.03c	0.73 ± 0.04a	0.58 ± 0.05a	0.74 ± 0.08a	0.46 ± 0.04b
	10–20	W1	0.54 ± 0.01a	0.61 ± 0.06a	0.58 ± 0.02a	0.39 ± 0.04b	0.56 ± 0.02b
		W2	0.61 ± 0.01a	0.61 ± 0.03a	0.89 ± 0.09a	0.59 ± 0.04a	0.60 ± 0.02b
		CK	0.58 ± 0.09a	0.56 ± 0.03a	0.69 ± 0.25a	0.42 ± 0.07b	0.75 ± 0.10a
	20–40	W1	0.38 ± 0.01a	0.31 ± 0.03b	0.37 ± 0.02a	0.36 ± 0.05b	0.44 ± 0.04b
		W2	0.40 ± 0.03a	0.44 ± 0.01a	0.39 ± 0.02a	0.46 ± 0.04a	0.65 ± 0.04a
		CK	0.42 ± 0.02a	0.43 ± 0.07a	0.41 ± 0.03a	0.37 ± 0.03b	0.60 ± 0.03a
Catalase	0–10	W1	1.85 ± 0.02b	1.73 ± 0.03b	1.84 ± 0.03a	1.81 ± 0.02a	0.94 ± 0.15a
		W2	1.88 ± 0.02b	1.80 ± 0.00a	1.87 ± 0.02a	1.82 ± 0.01a	0.96 ± 0.09a
		CK	1.92 ± 0.02a	1.73 ± 0.02b	1.87 ± 0.02a	1.82 ± 0.02a	0.96 ± 0.12a
	10–20	W1	1.82 ± 0.00b	1.95 ± 0.02a	1.92 ± 0.02a	1.85 ± 0.01a	0.94 ± 0.05a
		W2	1.84 ± 0.01a	1.96 ± 0.01a	1.93 ± 0.00a	1.87 ± 0.01a	1.04 ± 0.05a
		CK	1.84 ± 0.01a	1.96 ± 0.01a	1.94 ± 0.00a	1.84 ± 0.03a	1.02 ± 0.07a
	20–40	W1	1.64 ± 0.06b	1.65 ± 0.07b	1.50 ± 0.08c	1.60 ± 0.02b	0.44 ± 0.03b
		W2	1.78 ± 0.02a	1.82 ± 0.02a	1.76 ± 0.03b	1.83 ± 0.02a	0.57 ± 0.01a
		CK	1.80 ± 0.02a	1.84 ± 0.03a	1.92 ± 0.07a	1.85 ± 0.05a	0.54 ± 0.02a
Sucrase	0–10	W1	24.83 ± 2.79a	18.67 ± 1.33a	17.54 ± 1.07b	13.28 ± 1.06b	15.14 ± 1.94b
		W2	24.71 ± 2.12a	19.88 ± 3.39a	23.17 ± 1.03a	18.04 ± 1.53a	26.98 ± 1.99a
		CK	23.04 ± 2.51a	17.09 ± 1.79a	17.26 ± 0.00b	19.55 ± 1.41a	28.42 ± 2.47a
	10–20	W1	20.30 ± 1.78a	17.51 ± 1.79b	14.94 ± 0.58b	16.83 ± 0.87b	20.61 ± 1.01b
		W2	18.74 ± 0.27a	18.50 ± 0.59b	19.58 ± 1.70a	22.93 ± 0.86a	26.78 ± 1.57a
		CK	21.53 ± 2.12a	24.48 ± 1.87a	16.21 ± 0.60b	23.32 ± 1.99a	28.27 ± 1.10a
	20–40	W1	10.05 ± 1.88b	11.70 ± 1.34b	10.89 ± 1.18b	10.82 ± 0.62c	14.16 ± 0.85b
		W2	15.45 ± 1.77a	15.81 ± 1.78a	14.37 ± 0.36a	16.45 ± 1.56a	19.45 ± 0.45a
		CK	17.75 ± 2.34a	13.12 ± 0.84ab	13.06 ± 0.42a	14.16 ± 1.04b	18.50 ± 1.16a

Different lowercase letters indicate significant differences among treatments (*p* < 0.05). W1, Moderate water stress (55% of field capacity); W2, Mild water stress (65% of field capacity); and CK, Sufficient water supply (75% of field capacity).

### 3.3 Correlation analysis between soil physical–chemical and soil enzymes

**Figure 2:**
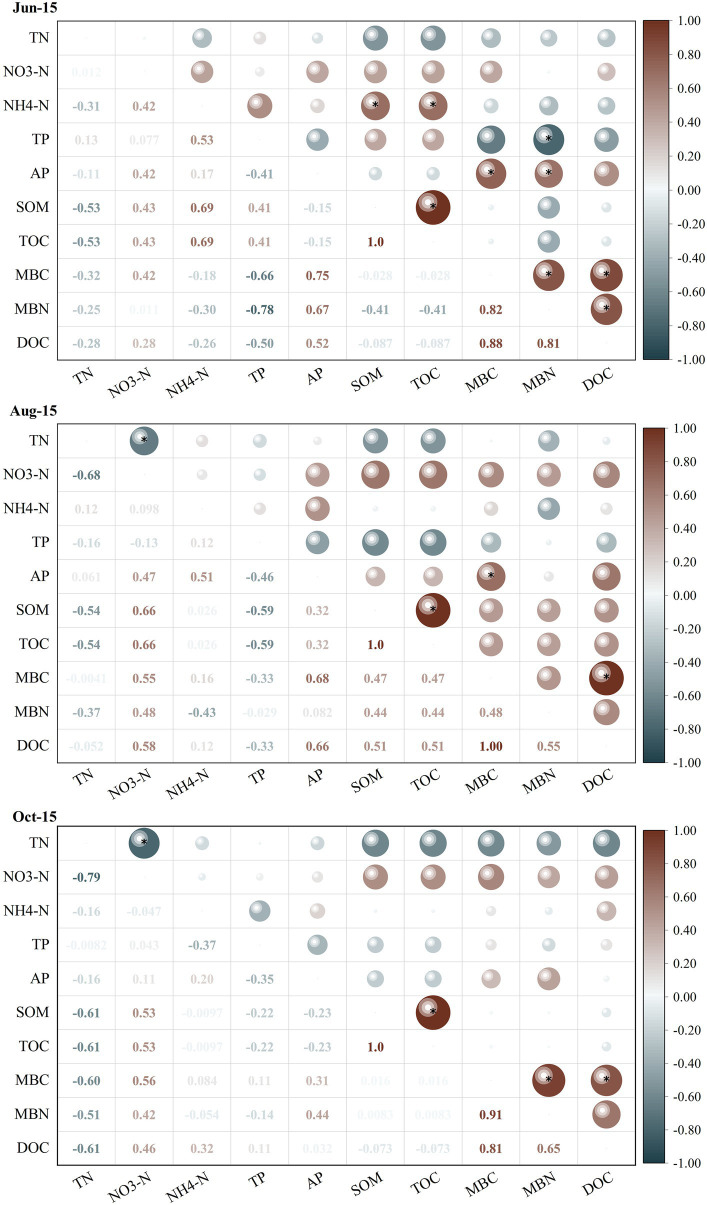
Correlation between soil physical–chemical indicators and microbial biomass indicators. ^*^Significantly correlated at the 0.05 level (two sides). TN, Total nitrogen; NO_3_-N, Nitrate nitrogen; NH_4_-N, Ammonia nitrogen; TP, Total phosphorus; AP, Availability phosphorus; SOM Soil organic matter; TOC, Total organic carbon; MBC, Microbial biomass carbon; MBC, Microbial biomass nitrogen; and DOC, Dissolved organic carbon.

**Figure 3:**
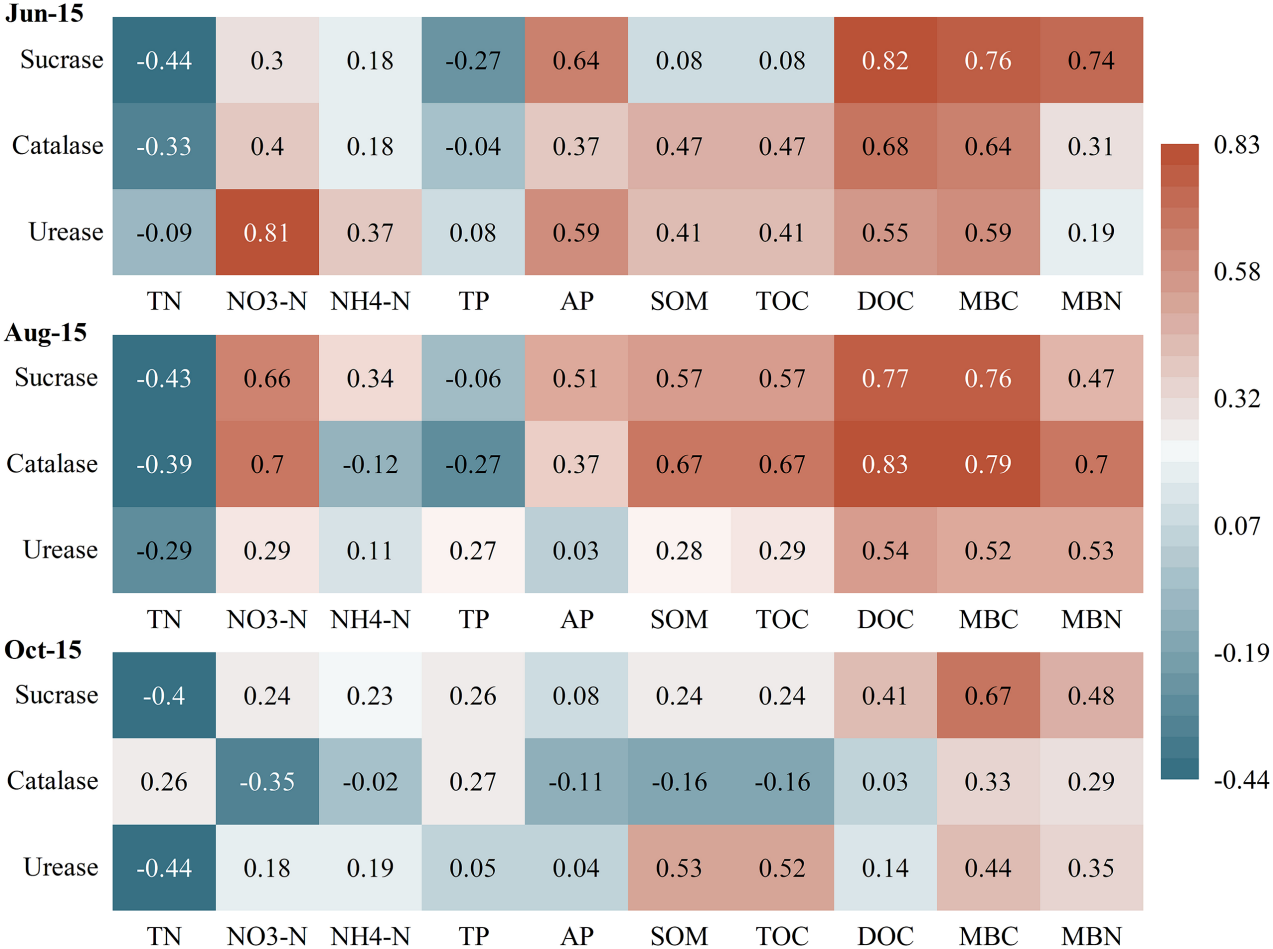
Correlation between soil physical–chemical indicators and soil enzymes. ^*^Significantly correlated at the 0.05 level (two sides). TN, Total nitrogen; NO_3_-N, Nitrate nitrogen; NH_4_-N, Ammonia nitrogen; TP, Total phosphorus; AP, Availability phosphorus; SOM, Soil organic matter; TOC, Total organic carbon; MBC, Microbial biomass carbon; MBC, Microbial biomass nitrogen; and DOC, Dissolved organic carbon.

The correlations between soil microbial biomass and basic soil physical–chemical indicators differed considerably between different growth periods of greenhouse grape rhizospheres (Figure 2). Among them, MBC and MBN showed significantly negative correlations (*r* = −0.67 and-0.78) with soil TP at the new growth stage (Jun-15) (*p* < 0.05), but significant positive correlations with soil AP and DOC (*p* < 0.05). In contrast, there was a highly significant positive correlation between MBC and DOC in the fruit enlargement stage (Aug-15) and coloring maturity stage (Oct-15). The correlation analysis between soil physical–chemical indicators and soil enzyme activities (Figure 3) showed that there were significant positive correlations between soil urease, catalase, and sucrase to varying degrees during the new growth stage (Jun-15), fruit enlargement stage (Aug-15), and coloring maturity stage (Oct-15). There were significant positive correlations between soil urease (Jun-15) and catalase (Aug-15) and nitrate nitrogen (*r* = 0.81, 0.70) (*p* < 0.05). Meanwhile, catalase showed a significant positive correlation between organic matter and TOC during the fruit enlargement stage (Aug-15). Soil enzyme activity was positively correlated with DOC, MBC, and MBN at different stages.

## 4 Discussion

The results of this study indicated that TN, NH_4_-N, and TP content in grape rhizosphere soil had the similar response to water stress, without the significant difference among three treatments (moderate and mild water stress and adequate water supply). The results were consistent with previous finding that soil TN and TP contents were not sensitive under different water stress (Wu et al., 2012; Bu et al., 2018). In addition, the study also found that the effect of water stress on soil NO_3_-N and AP contents were closely related to the degree and time of stress because the soil NO_3_-N and AP contents were significantly lower under moderate (W2) and mild (W1) water stress than with sufficient water (CK) in the new shoot elongation stage (Jun-15) and fruit enlargement (Aug-15) stages. Water stress reduced the vitality of the plant’s root system, which in turn decreased the secretion of all kinds of organic and inorganic substances by the plant’s root system (Draye et al., 2010). On the contrary, in the coloring maturity stage (Oct-15), there was no significant difference in the NO_3_-N and AP contents of grape rhizosphere soil under continuous water stress (Li et al., 2021). The cause was due to the decrease of nutrient uptake by the crop’s root system as the grapes mature. However, the result showed stronger selective absorption of nitrate nitrogen compared with NH_4_-N, as well as the promotion of the absorption of AP content in the soil under water stress.

Another important finding was that there was no significant effect on soil TOC content in the rhizosphere of grapes under water stress during the growth period. A possible explanation may be that the accumulation in soil organic carbon pool storage was a relatively long process, and the change in the amount of grape root exudates caused by water stress was not enough to cause an obvious change in the soil organic carbon content. Additionally, the grape rhizosphere exudates were first supplied to rhizosphere soil microorganisms for utilization and reproduction (Song et al., 2012). Therefore, water stress had no significant effect on the soil TOC content; during the growth period had small fluctuations. Meanwhile the soil DOC content significantly reduced. The soil DOC content under moderate water stress was significantly lower than that under mild water stress and for control treatment with the promoted degree of water stress (W2 and W1 treatments) and the prolonged stress time. This finding was contrary to previous results that suggested DOC could increase with the decline of soil moisture (Wang et al., 2017). The results of the present study showed that moderate water stress reduced the DOC content of grape rhizosphere soil, but mild water stress had no significant effect. This finding was also reported by Li et al. (2016). In summary, soil carbon content may be related to a number of factors such as geographical location, field management, and irrigation practices (Zhang R. et al., 2023). However, water stress reduced the soil MBC and MBN contents of the grape rhizosphere soil, which was contrary to previous findings that drought stress increased the MBC content (Mganga et al., 2019). Previous studies also suggested that excessive water stress (Hueso et al., 2012) and long-term water stress also reduced the soil microbial biomass content (Schimel et al., 1999; Wang et al., 2017). This could be explained by the fact that water stress reduced the vitality of plant roots, which in turn caused the reduction of various organic and inorganic substances secreted by plant roots. This finding was contrary to previous results suggesting that water-soluble compounds and mucilage secreted by plant roots under drought stress promoted the production of microbial biomass (Sanaullah et al., 2011).

Soil moisture was an important factor affecting soil enzyme activity in plant rhizosphere (Geisseler et al., 2011; A'Bear et al., 2014). This experimental study showed that the soil urease activity increased during the initial period of water stress and decreased significantly in the mid-stress stage in soil layers of 0–10 and 10–20 cm, which was probably varied in different plant growth period (Song et al., 2012; Zhang et al., 2018). With the increase of stress duration, moderate water stress reduced the soil urease activity in the soil layer of 0–10 cm, and the moderate and mild water stress reduced the soil urease activity in the soil layer of 40 cm during the coloring mature stage. Adequate water supply and mild water stress helped increase the urease activity in the soil layer of 20–40 cm. Another finding was that in terms of spatial distribution, the activity of urease, catalase, and sucrase in greenhouse grape rhizosphere soil in different soil layers followed the order: 10–20 cm > 0–10 cm > 20–40 cm. This result might be explained by the fact that fertilizers (organic fertilizer, farmyard manure, and chemical fertilizer) were sprayed onto the soil trough 20 cm away from the grape roots and then covered with topsoil (Liu et al., 2024). The urease and sucrase activities in rhizosphere soil were basically stable during the whole growth period of grapes. Hence, further studies should be conducted to investigate the growth, yield, and quality of grapes under different water stresses, and to provide a better description of the influence of water regulation on the water-rhizosphere soil–plant system. In addition, mild water stress helped increase the catalase activity in grape rhizosphere soil in the soil layer of 0–10 cm on Jun-15 (new shoot growth stage) and Aug-15 (fruit expansion stage). On May-15 (budburst stage), the soil catalase activity under moderate water stress was significantly lower than that with an adequate water supply and under mild water stress. Soil catalase activity was related to various factors such as soil fertility, texture, pH, aeration, and climatic conditions (Bao et al., 2022). This experimental study found that W2 treatment helped increase the soil sucrase activity in the soil layer of 0–10 cm on July 20 (flowering stage), but it reduced the soil invertase activity from May-15 (budburst stage) to Jun-15 (new shoot growth stage). This was because the soil conditions between the roots may become anaerobic microdomains due to reduced water content, and could lead to the inhibition of sucrase activities because of limited substrate diffusion and oxygen content (Zhang, 2005).

## 5 Conclusion

This study detected significant differences in rhizosphere soil enzyme activities and microbial biomass of greenhouse grape under water stress (*p <* 0.05). Moreover, water stress had less effect on soil physical–chemical properties. Compared with the adequate water supply conditions, the water stress (mild W2 and moderate W1) effectively reduced the accumulation of soil MBC content throughout the grape growing season and reduced soil MBN content in later growth. Both W2 (mild water stress) and W1 (moderate water stress) treatments inhibited the activities of urease, catalase, and sucrase activities transforming enzymes in the soil during greenhouse grape growth. These results illustrated that water stress altered both soil microbial structure and function in rhizosphere soil enzyme activities. Overall, this study provides a theoretical basis for water-saving greenhouse grape cultivation and soil environment regulation. Considering the effects of successive years of water stress on soil microbial and rhizosphere enzyme activities in greenhouse grapes needs to be further deepened and broadened. We suggest that future studies should focus more on changes in greenhouse grape rhizosphere soil enzyme activities and microorganisms as a result of multi-year water stress, and should incorporate changes in microbial communities, which play a crucial role in the regulation of soil quality and plant acclimatization.

## Data availability statement

The original contributions presented in the study are included in the article/supplementary material, further inquiries can be directed to the corresponding author.

## Author contributions

RZ: Funding acquisition, Writing – review & editing. HZ: Data curation, Writing – original draft. CY: Data curation, Writing – review & editing. HL: Investigation, Writing – review & editing. JW: Formal Analysis, Methodology, Writing – review & editing.
